# PORTAL VEIN THROMBOSIS AFTER IATROGENIC ENDOSCOPIC BILIARY PROSTHESIS PLACEMENT

**DOI:** 10.1590/0102-6720202400038e1832

**Published:** 2024-11-15

**Authors:** Sterphany Ohana Soares Azevêdo PINTO, Marcelo Olivati do AMARAL, Angelo So Taa KUM, Marcos Eduardo Lera dos SANTOS, Ralph Rodrigo Francisco Martins TAVARES, Luiz Augusto Carneiro D’ALBUQUERQUE, José JUKEMURA, André Luis MONTAGNINI

**Affiliations:** 1 Universidade de São Paulo, Faculty of Medicine, Hospital Universitário, Department of Gastroenterology - São Paulo (SP), Brazil

## INTRODUCTION

Endoscopic stent placement has become a well-established treatment in the management of biliary tract obstruction^8^. Complications such as pancreatitis, hemorrhage, duodenal perforation, or cholangitis can occur in up to 10% of insertions^4,6,8^. Iatrogenic perforation of the portal vein during the procedure or stent migration to the portal system are unusual complications, and due to scarce reports in the literature, there is no consensus on how to manage it^1,2,11^.

Here, we present a case of portal thrombosis secondary to an accidental portal insertion of a biliary stent. The study was approved by the Ethics Committee of the Institution (number 76693423.1.0000.0068).

## CASE REPORT

A 44-year-old female presented in another medical institution with acute upper abdominal pain, jaundice, and vomiting, and was diagnosed with mild acute biliary pancreatitis. Magnetic resonance imaging (MRI) showed choledocholithiasis. As the jaundice persisted, she underwent endoscopic retrograde cholangiopancreatography (ERCP). After undergoing papillotomy and plastic stent placement, the clinical jaundice subsided. The clinical report did not mention technical difficulties.

After 4 months, the patient was referred to our outpatient facility due to upper abdominal pain and generalized pruritus. Blood tests showed: total bilirubin, 2.06 mg/dL (reference range, 0.2-1.2 mg/dL); direct bilirubin, 1.0 mg/dL (up to 0.5 mg/dL); indirect bilirubin, 1.1 mg/dL (0.2-0.7 mg/dL); alkaline phosphatase, 270 U/L (40-150 U/L), and gamma-glutamyl transferase, 431 U/L (12-64U/L).

**Figure 1:**
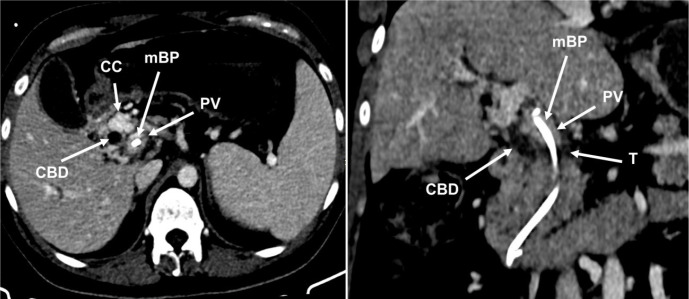
Contrast-enhanced computed tomography showing the proximal end of the biliary stent inside the portal vein and portal vein cavernous transformation.

CBD: common bile duct; CC: collateral circulation; mBP: malpositioned biliary prosthesis; PV: portal vein; T: tumor at the pancreatic head.

**Figure 2:**
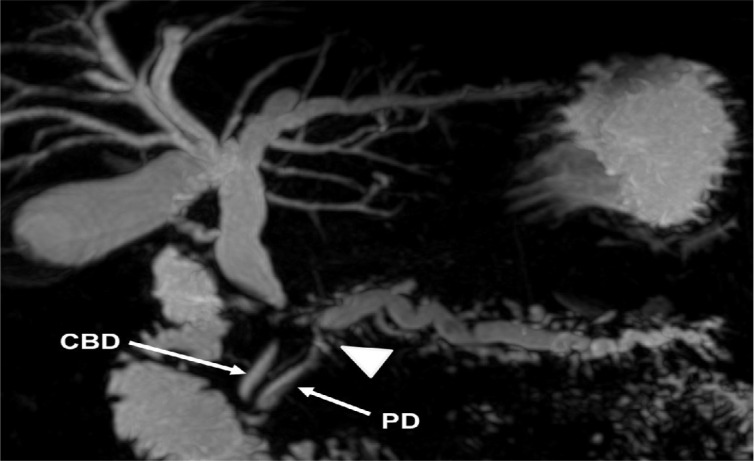
Magnetic resonance cholangiopancreatography showing biliary and pancreatic duct dilation and distal tapering of the common bile duct.

CBD: common bile duct; PD: pancreatic duct. The arrowhead indicates where the biliary prosthesis might have perforated the pancreatic duct, thus eventually being inserted in the portal vein. Note that the pancreatic duct is dilated right after this point.

Contrast-enhanced computed tomography (CT) showed a portal vein cavernous transformation, a mass lesion in the head of the pancreas, and that the proximal end of the stent was inside the portal vein (Figure 1). Magnetic resonance cholangiopancreatography showed biliary and pancreatic duct dilation (Figure 2). Through endoscopic ultrasound, the fine needle aspiration identified only inflammatory infiltrates. Tumoral markers identified were as follows: carbohydrate antigen 19.9, 18.4 U/mL (up to 37 U/mL) and carcinoembryonic antigen, 1.73 ng/mL (up to 5 ng/mL).

In a multidisciplinary discussion, we considered that the risk for malignant disease was low, and that, although there was portal vein thrombosis, the cavernomatous transformation could yield a risk for abundant bleeding if the prosthesis were to be removed. However, as the patient had clinical signs of stent malfunction and imaging showed a dilated biliary system, the misplaced stent had to be removed and the biliary duct had to be correctly drained. We decided to withdraw the stent endoscopically in an operating room, with interventional radiologists and surgeons prepared to step in in case of substantial bleeding.

**Figure 3:**
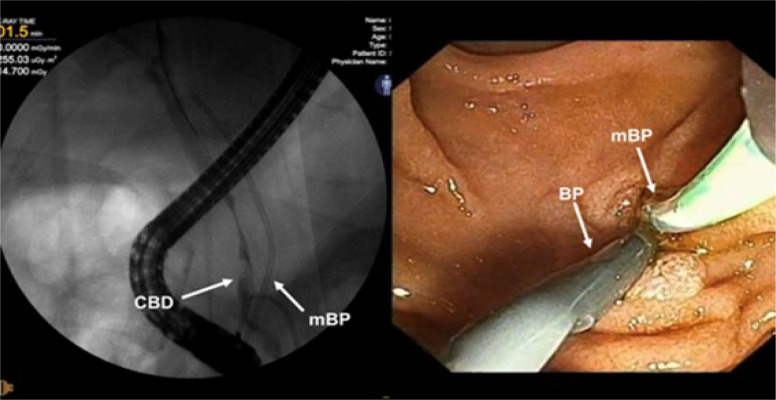
Endoscopic retrograde cholangiopancreatography and endoscopy showing the two prostheses, right before the removal of the mispositioned stent.

CBD: common bile duct; mBP: malpositioned biliary prosthesis; BP: new biliary prosthesis.

Through ERCP, the previous papillotomy was extended, and a new plastic endoprosthesis (8.5 Fr × 10 cm) was placed inside the biliary duct (Figure 3). Bile and contrast were successfully drained through the stent. We then removed the misplaced stent, and at this point, there was mild self-resolving bleeding.

After the procedure, abdominal pain and pruritus progressively subsided. Blood workup showed normalization of bilirubin. During a 10-month follow-up, the patient remained asymptomatic.

## DISCUSSION

The first report of portal injury during an endoscopic attempt to drain the biliary tract was described by Huibregtse et al. in 1988^4^. A venous puncture was suspected when fluoroscopy showed filling of the venous system. While fluoroscopy is an important tool, it may not be sufficient to avoid dismissing complications. Ricci et al.^8^ and van Buuren et al.^10^ reported cases where the portal system was interpreted to be the biliary tract during fluoroscopy. It was hypothesized that the anatomic similarity between the two systems and a short fluoroscopy time were major factors that contributed to the confusion^8,10^. They suggested that the successful aspiration of bile, besides the correct recognition of the anatomic pattern of the biliary system, is also an important measure to ensure procedural safety^10^. For the presented case, the possible mechanism for the misplacement was that, after transpapillary access, the guidewire entered the pancreatic duct and, at the level of a stenosis, transfixed the pancreatic parenchyma and reached the portal vein, mimicking the biliary tree anatomy during fluoroscopy.

In almost all cases reviewed, a difficult endoscopic procedure was reported^1,2,3,7-10^. Difficult cannulation, the need for sphincterotomy, and resistance during biliary access were often described^1,2,9^. Our patient had undergone ERCP shortly after an episode of pancreatitis, and local inflammation was probably an important component in the subsequent complication.

Most reported cases had an immediate (during the procedure) or early (within 1 week of the procedure) onset of symptoms^2,3,5,7-10^. When immediate, self-resolving hemobilia or blood on suction after cannulation were the most common manifestations^3,5,8,10^. When early or delayed onset, most patients presented gastrointestinal bleeding, sometimes life-threatening^1-3,7,11^. Delayed presentation is most likely linked to proximal stent migration, and not perforation during endoscopy. Still, even though our patient had symptoms 4 months after the first ERCP, we believe that the fistula was caused during prosthesis insertion, considering the abundant collateral circulation and absence of gastrointestinal bleeding.

Treatment options reported varied from surgical and endoscopic removal of the stent to conservative management, especially for palliative care patients^3^. None of the reviewed studies reported early or late poor outcomes. Endoscopic treatment in an operating room is an option that allows for the use of a least-invasive approach while having a set scenario for any intercurrences.
